# ES-Screen: A Novel Electrostatics-Driven Method for Drug Discovery Virtual Screening

**DOI:** 10.3390/ijms232314830

**Published:** 2022-11-27

**Authors:** Naiem T. Issa, Stephen W. Byers, Sivanesan Dakshanamurthy

**Affiliations:** Department of Oncology, Lombardi Comprehensive Cancer Center, Georgetown University Medical Center, Washington, DC 20057, USA

**Keywords:** electrostatics, electrostatic potential, electrostatic energy, free energy, virtual screening, hit-to-lead identification, drug discovery

## Abstract

Electrostatic interactions drive biomolecular interactions and associations. Computational modeling of electrostatics in biomolecular systems, such as protein-ligand, protein–protein, and protein-DNA, has provided atomistic insights into the binding process. In drug discovery, finding biologically plausible ligand-protein target interactions is challenging as current virtual screening and adjuvant techniques such as docking methods do not provide optimal treatment of electrostatic interactions. This study describes a novel electrostatics-driven virtual screening method called ‘ES-Screen’ that performs well across diverse protein target systems. ES-Screen provides a unique treatment of electrostatic interaction energies independent of total electrostatic free energy, typically employed by current software. Importantly, ES-Screen uses initial ligand pose input obtained from a receptor-based pharmacophore, thus independent of molecular docking. ES-Screen integrates individual polar and nonpolar replacement energies, which are the energy costs of replacing the cognate ligand for a target with a query ligand from the screening. This uniquely optimizes thermodynamic stability in electrostatic and nonpolar interactions relative to an experimentally determined stable binding state. ES-Screen also integrates chemometrics through shape and other physicochemical properties to prioritize query ligands with the greatest physicochemical similarities to the cognate ligand. The applicability of ES-Screen is demonstrated with in vitro experiments by identifying novel targets for many drugs. The present version includes a combination of many other descriptor components that, in a future version, will be purely based on electrostatics. Therefore, ES-Screen is a first-in-class unique electrostatics-driven virtual screening method with a unique implementation of replacement electrostatic interaction energies with broad applicability in drug discovery.

## 1. Introduction

Electrostatics interactions are important drivers of biomolecular recognition [1,2], associations [3,4], and kinetics [5,6,7,8]. These interactions are important determinants of ligand binding affinity and can serve to discriminate between active and non-active chemical congeners in the “hit-to-lead” drug development process [9]. Treatment of electrostatics in in silico screening has been a contentious issue. Current virtual screening protocols utilize docking to calculate the free energy of binding and determine binding poses with low computational cost [10]. Some docking algorithms incorporate molecular mechanics and treatment of polar and non-polar contributors to binding free energy and generally successfully provide initial ligand hits. Notably, performance varies across different protein targets and the chemical library being screened [11,12]. Free energy calculations occur in vacuo devoid of solvent effects, and electrostatics is generally treated as the distance-dependent interactions between ligand and protein point charges represented by Coulomb’s Law. In addition, the electrostatics component may not be weighted appropriately for free energy calculations.

More accurate computational methods for estimating free energies of binding have emerged that solve the Poisson-Boltzmann equation (PBE), which incorporates an implicit solvent model and accounts for ionic effects [13]. These methods include PBSA and GBSA protocols [14], which calculate polar and non-polar solvation-free energies while incorporating molecular mechanics. While these calculations are more accurate than docking [15], they are still not amendable to traditional virtual screening protocols as they require pre-docked poses and integrate protein/ligand flexibility. Accuracy is therefore affected by docking pose and molecular dynamics simulations. Furthermore, PBE solvers vary in methodology [16] for calculating electrostatic potentials (ESP), giving rise to additional inconsistencies.

**Figure 1:**
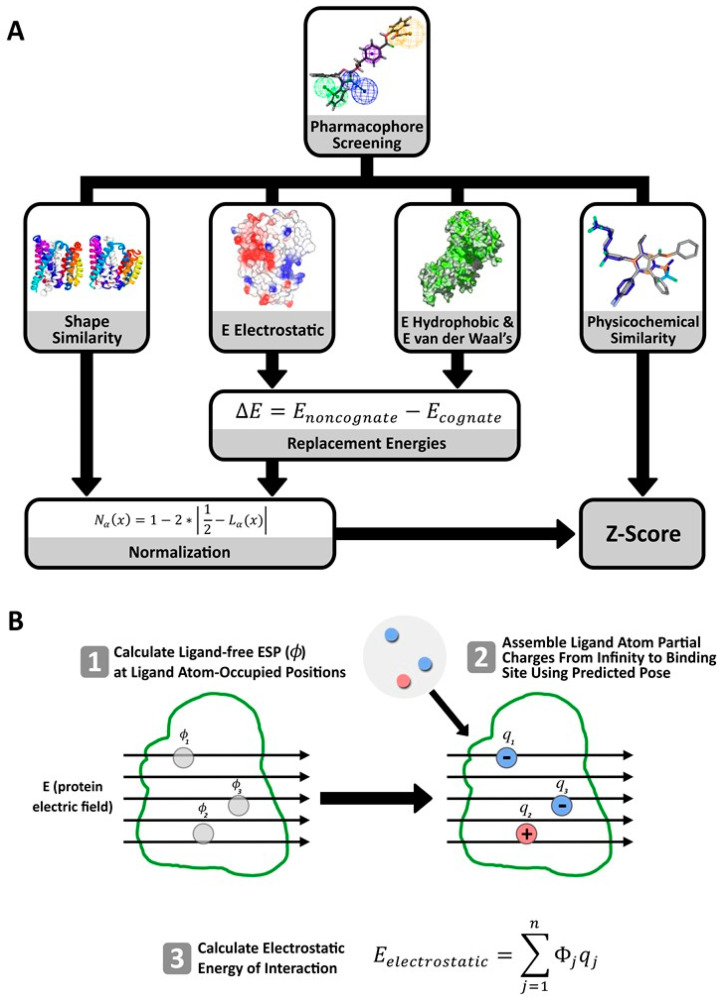
(**A**) Schematic of ES-Screen for predicting ligand-target interactions. (**B**) Process of determining the electrostatic energy of interaction. Using predicted ligand-protein binding poses after pharmacophore screening: (1) ligand-free electrostatic potentials (ESP, *Φ*) given by the protein electric field (E) are calculated using Delphi at positions occupied by ligand atoms, (2) ligand atoms, represented as partial charges, are then assembled within the pocket from infinity within solvent of high dielectric constant to ligand-occupied positions within the solute of low dielectric constant, and (3) the electrostatic energy of interaction is calculated using Equation (1).

We describe a novel electrostatics-driven method, entitled ES-Screen (Figure 1), to provide greater enrichment in virtual screening experiments over docking and GBSA/PBSA methods. We performed preliminary ES-Screen benchmarking using the DUD-E [17] chemical set for its carefully curated decoys. ES-Screen was then assessed across 53 unique protein targets using a diverse chemical library containing over 3600 FDA-approved and experimental drugs. The FDA-approved and experimental drugs were obtained from the ZINC database (https://zinc.docking.org/ (accessed on 4 October 2014). ES-Screen was further used to discover novel kinase (PI3KCG, JAK2, RAF1, MNK2) [18,19,20,21] and other targets such as FABP4 and Aldose Reductase for the anti-hookworm medication mebendazole (MBZ), verified in in vitro binding assays, providing further impetus for its repurposing as an anti-cancer agent. We also confirmed that ES-Screen identified new targets FABP4 and Aldose Reductase for Licofelone, Indomethacin, and Oxaprozin.

ES-Screen is the first-in-class unique electrostatics-driven method that performs accurate virtual screenings to identify novel ligand-target binding interactions. The incorporation of replacement energies, in particular electrostatic interaction energies, allows for high-fidelity molecular discrimination. Coupled with its low computational cost compared to other virtual screening methods, ES-Screen can serve as a holistic in silico platform for de novo drug discovery.

## 2. Results

### 2.1. ES-Screen Method

We developed a novel virtual screen method called ‘ES-Screen’ (Figure 1A). The ES-Screen is independent of docking because it utilizes knowledge-based pharmacophore hypotheses derived from ligand-protein binding complex structures for optimized pose prediction. Electrostatic interaction energies are calculated through a two-step process (Figure 1B). The ligand-free protein ESP has extrapolated to ligand atom-occupied positions within the binding site, and ligand atom partial charges (Figure 1B) are subsequently assembled from the solvent with a high dielectric constant into the binding site with a low dielectric constant as described previously by Dakshanamurthy et al. and Basu et al. [1,2]. ES-Screen energetically prioritizes molecules using the energy cost of replacing the cognate ligand with the non-cognate molecules being screened, where negative energies are more favorable. The electrostatic component of replacement energies is the energy costs of placing a query (non-cognate) ligand at the binding site in place of the reference (cognate) ligand molecule. The use of individual replacement energies in the ES-Screen is a novel strategy and differs from a ranking based on the predicted free energy of binding, which is first calculated by combining all components. Dakshanamurthy et al. and Basu et al. previously employed this unique electrostatic treatment to discriminate between ATP and GTP binding proteins [1,2]. The detailed procedure of ESP extrapolation, and the calculation of electrostatics replacement energy are described by Dakshanamurthy et al. and Basu et al. previously [1,2]. The complete ES-Screen methodology, including calculating various energy terms, is described in the methods section.

The ES-Screen also treats non-polar contributions by calculating individual hydrophobic and van der Waal’s solvation energies. ES-Screen then incorporates protein-ligand shape-, and physicochemical-based features that leverages information derived from protein-ligand co-crystal structures and comprehensively model the binding interaction space (Figure 1A). In addition, ES-Screen uses a knowledge-based approach, and prioritizes molecules that exhibit the greatest shape and physicochemical similarity to the reference (cognate) ligand. The use of replacement energies with respect to each contributor of binding (electrostatic, hydrophobic, van der Waal’s) optimizes for thermodynamically favorable ligands across all categories concomitantly. To provide a final ranking score (Equation (5)), individual replacement energy terms are then combined with the one-dimensional shape and physicochemical fingerprint similarities. The electrostatics term is given preferential weight over non-polar terms.

Before energy calculations, ligands are first placed into putative binding pockets through knowledge-based pharmacophore models. Such models are derived from crystal structures to optimize ligand poses by shape and functional group orientation. Application of excluded volume spheres further refines ligand pose by minimizing ligand-receptor atomic clashes. Subsequently, single-point interaction energies were individually calculated for electrostatic, hydrophobic, and van der Waals contributions to the binding. These values were then subtracted from those of the co-crystallized reference (cognate) ligand to obtain replacement energies, which have been implicated in high-fidelity molecular discrimination [1,2]. After normalization, these values were combined with the shape and other ligand descriptor similarity terms (chemometrics) to give a Z-score for ranking ligands based on the likelihood of binding to the putative protein target conformation state.

### 2.2. Initial Performance Assessment of ES-Screen Using DUD-E Benchmarking Set

**Figure 2:**
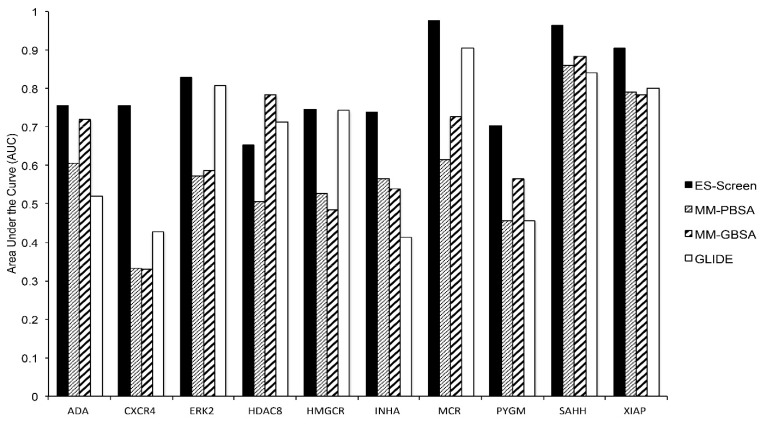
Areas under the curve for the DUD-E dataset determined from ROC curves in Figure 1 include MM-GBSA/PBSA methods.

**Figure 3:**
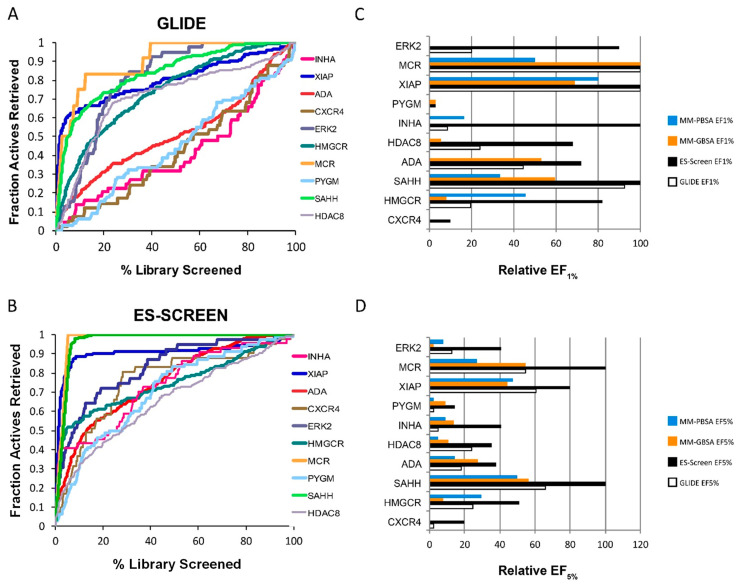
Virtual screening performance using DUD-E benchmarks. (**A**,**B**) Receiver operating characteristic (ROC) curves for (**A**) GLIDE-SP docking and (**B**) ES-Screen. (**C**,**D**) Relative enrichment factors of (**C**) the top 1% and (**D**) the top 5% for GLIDE-SP docking, MM-PBSA, MM-GBSA, and ES-Screen.

The DUD-E dataset [17] was first used to assess the performance of ES-Screen. This set was chosen for its minimal decoy bias while maintaining ligand diversity. In particular, DUD-E provided chemical sets with high structural similarity but differing net charges, allowing for the exploitation of electrostatic interactions. Ten protein targets with pharmaceutical precedence encompassing multiple protein families were chosen for virtual screening. ES-Screen substantially improves performance over GLIDE-SP docking, MM-GBSA, and MM-PBSA methods in prioritizing known actives (Figure 2 and Figure 3). When considering performance through receiver operating characteristic (ROC) curves, ES-Screen performs better than chance and increases the area under the curve (AUCs) for 9 out of 10 protein targets (Figure 3A,B). ES-Screen also produces the greatest relative enrichment factors (EFs) at 1% (Figure 3C) and 5% (Figure 3D) compared to all three methods, implying the potential universal applicability of ES-Screen with minimized false positivity.

### 2.3. ES-Screen Performance Using Approved and Experimental Drugs

**Figure 4:**
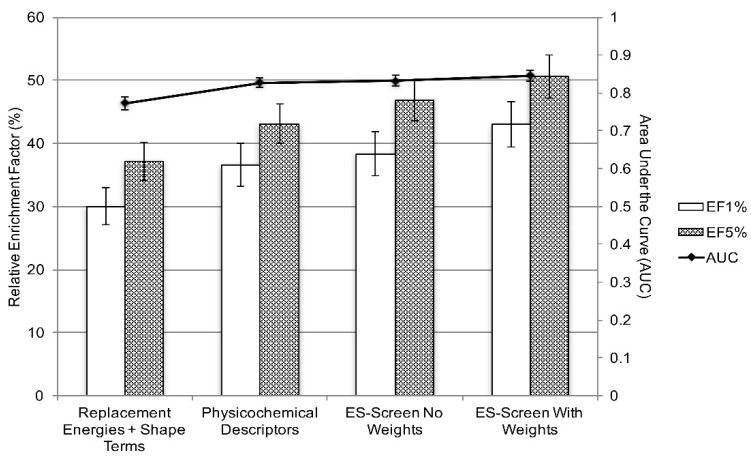
Improvement of virtual screening performance with a combination of parameters and weight adjustment in building ES-Screen. Average performance across N = 53 protein targets using relative enrichment factors EF1% and EF5% and areas under the curve (AUC) derived from ROC curves. Error bars correspond to standard errors of the mean.

Next, ES-Screen performance was gauged using chemical datasets containing over 3600 approved and experimental drugs on a larger protein target dataset (N = 53). Virtual screenings for the prioritization of known drug binders obtained from DrugBank and RCSB were performed. ES-Screen parameters and weights, initially used on the DUD-E datasets, emphasizing the replacement electrostatic interaction energy, produced optimal enrichment (Figure 4). Maximum area under the curve (AUC) and relative enrichment factors for both the top 1% (EF1%) and 5% (EF5%) were achieved using ES-Screen as described by Equation (5) in the Methods section (Figure 4).

**Figure 5:**
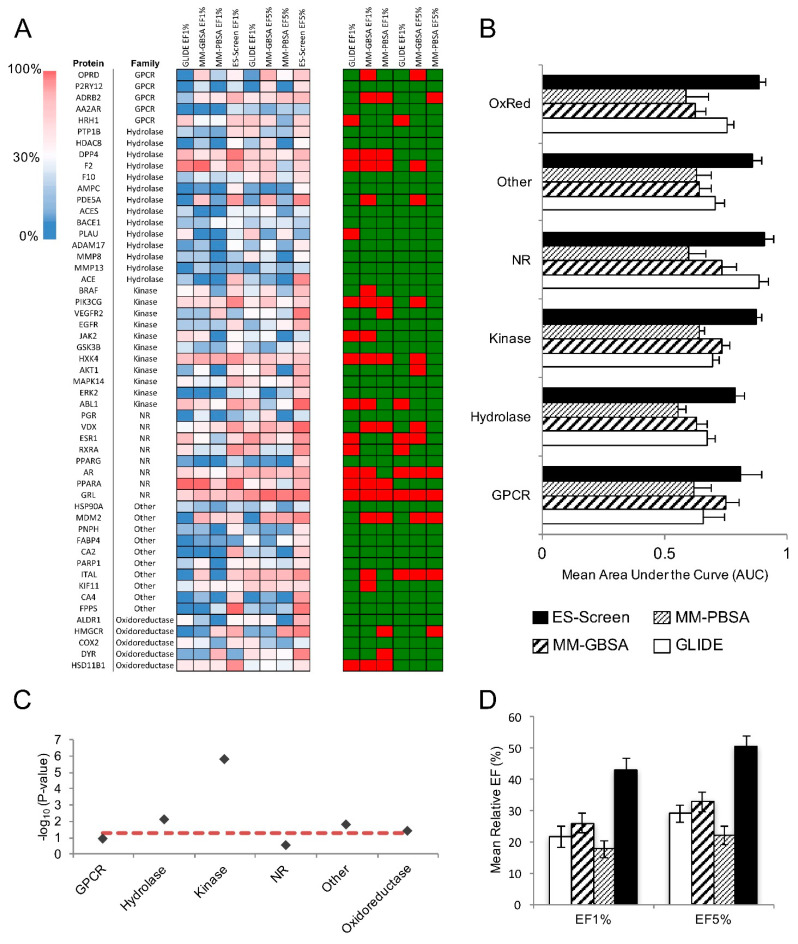
ES-Screen performance compared to GLIDE-SP for virtual screenings using a chemical dataset of FDA-approved and experimental drugs. (**A**) Heatmap of relative enrichment factors (EF1% and EF5%) for individual protein targets with respect to each virtual screening method; colored boxes for EF1% improvement and EF5% improvement represent whether ES-Screen performs superiorly (green box) or inferiorly (red box) with respect to each method; (**B**) Performance measured using the mean area under the curve (AUC), obtained from ROC curves, within each protein family, error bars represent standard errors of the mean (abbreviations: GPCR, G protein-coupled receptor; NR, nuclear receptor; OxRed, oxidoreductase; *p*-value < 0.05 on one-tailed *t*-test with respect to ES-Screen). (**C**) Log-transformed *p*-values of one-tailed t-tests comparing the ES-Screen to GLIDE docking, red dashed line represents statistical significance level at *p* < 0.05. (**D**) Mean relative enrichment factors for the top 1% and 5% of the database screened across all protein targets (N = 53) are depicted; significance at *p* < 0.01 and *p* < 0.05 reported using a paired, one-tailed student’s *t*-test with respect to ES-Screen (black bar).

**Figure 6:**
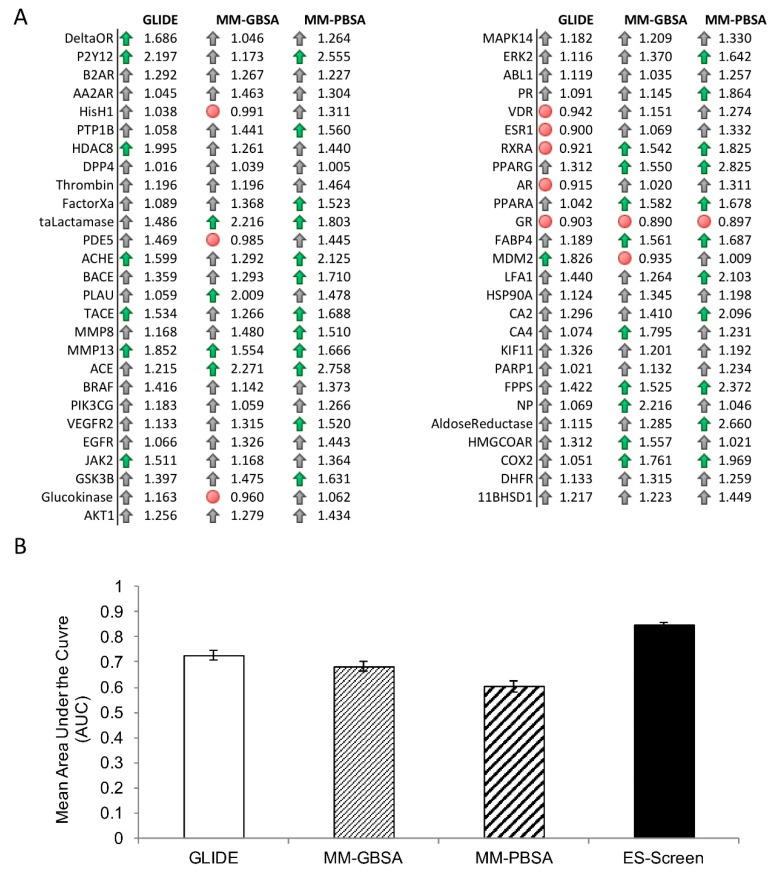
(**A**) Ratios of the areas under the curves (AUCs) obtained from ROC curves between ES-Screen and the indicated methods with respect to each protein target. Green arrows correspond to ES-Screen providing a >1.5-fold improvement, grey arrows correspond to ES-Screen providing <1.5-fold improvement, and red circles indicate instances where ES-Screen performed inferiorly to the respective method. (**B**) Bar graph of the mean AUCs across all protein targets with respect to each virtual screening method.

ES-Screen performed superiorly to GLIDE-SP docking, MM-GBSA, and MM-PBSA methods for most protein targets with respect to EF1% and EF5% (Figure 5). Improvement in AUCs was remarkable for many targets, exceeding 1.5-fold improvement (Figure 6A). ES-Screen performed worse in only 5, 5, and 1 protein targets than GLIDE-SP, MM-GBSA, and MM-PBSA, respectively. The performance also differed across protein families (Figure 5B). ES-Screen resulted in mean AUCs greater than 0.75, whereas all other methods resulted in mean AUCs less than 0.75 for each protein family (Figure 5B) and across all targets collectively (Figure 6B). The best performance for either virtual screening method was found for the nuclear receptor family, with both methods performing comparably (Figure 5B). Statistically significant improvements (one-tailed *t*-test, *p* < 0.05) were achieved for ES-Screen for protein families such as hydrolases, kinases, nuclear receptors, GPCRs, and oxidoreductases, with kinases achieving the greatest significance (Figure 5C). Notably, the GPCR family exhibited the greatest variability in performance, with ES-Screen missing statistical significance against GLIDE-SP and MM-GBSA in the face of the greatest mean AUC improvement (Figure 5B,C). This discrepancy is expected as GPCRs exhibit several binding modes within a large solvent-exposed pocket [19]. Overall, ES-Screen provided a statistically significantly more significant enrichment in performance over Glide docking, MM-GBSA, and MM-PBSA for the entire protein target population studied (Figure 5D). When considering relative EF1% and EF5%, ES-Screen provided an average increase in enrichment of 22.50% and 23.76%, respectively, over docking, 18.70%, and 20.84% over MM-GBSA, as well as 26.47% and 30.82% over MM-PBSA (Figure 5D). ES-Screen, therefore, is applicable as an all-purpose virtual screening method across a diverse set of protein targets.

**Figure 7:**
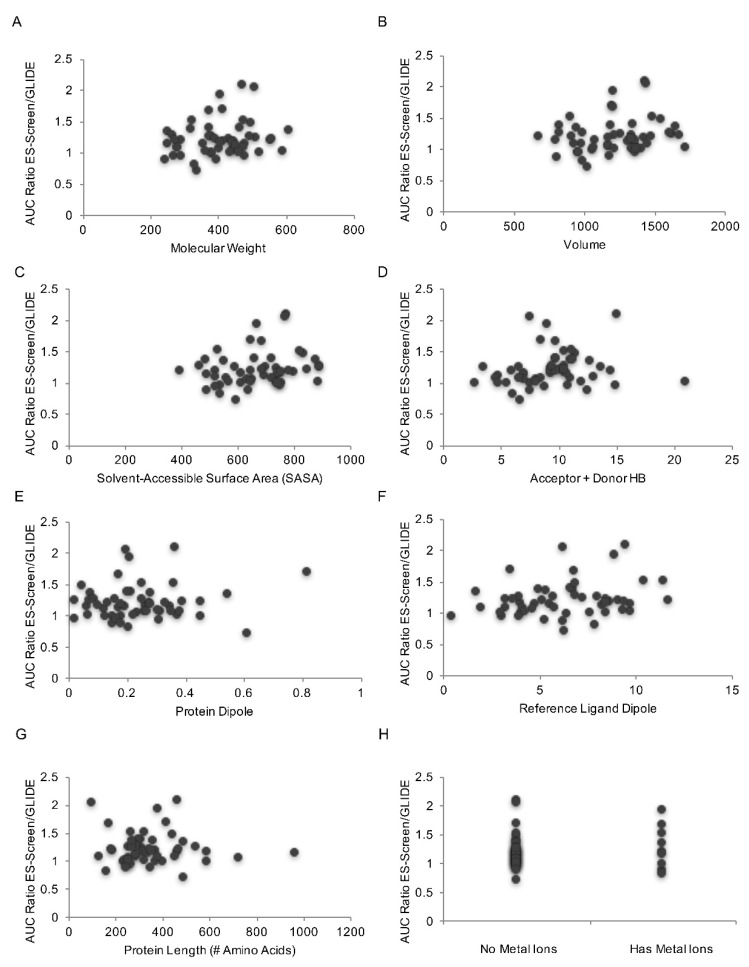
Trends in ES-Screen performance with respect to reference ligand and protein target physicochemical properties. Scatter plots of relative ES-Screen to GLIDE-SP docking performance for each protein target were studied against the following parameters: (**A**) reference ligand molecular weight, (**B**) reference ligand volume, (**C**) reference ligand solvent-accessible surface area (SASA), (**D**) reference ligand combined number of potential acceptor/donor hydrogen bonds, (**E**) protein dipole moment, (**F**) reference ligand dipole moment, (**G**) length of protein determined by a number of amino acids, and (**H**) presence of metal cations within the protein.

We further hypothesized that the increase in performance of ES-Screen with respect to docking would correlate with protein or reference ligand properties that are sensitive to the magnitude and directionality of molecular charges and macromolecular size. No correlations were observed for the following reference ligand properties: molecular weight, solvent-accessible surface area, volume, and the combined number of potential H-bonds donated/accepted (Figure 7A–D). Notably, no trends were observed when considering protein or reference ligand dipoles, several amino acids, and whether targets contain metal cations in the binding pocket (Figure 7E,F). These outcomes imply that ES-Screen provides consistent performance enrichment over docking across a variety of protein-reference ligand complexes that exhibit electronic and physicochemical diversity.

### 2.4. Experimental Validation of ES-Screen Predictions

#### 2.4.1. Mebendazole Binds Previously Unreported Protein Kinases

ES-Screen was used to predict novel kinase targets for the anti-hookworm medication mebendazole (MBZ), a tubulin inhibitor with anti-cancer potential [21] and a desirable toxicity profile. MBZ was predicted to bind PI3KCG, JAK2, RAF1, and MNK2 kinases. In vitro binding assays confirmed these interactions (Table 1). The newly defined kinase targets for MBZ at sub-micromolar affinity are also important cancer targets. MBZ has been studied for many cancers, including GBM [22], lung [23], and colon [24]. Although other benzimidazole drugs have been studied for hematologic malignancies [25], mebendazole has not yet been assessed. The anti-helminthic drug mebendazole has been previously determined to have anti-cancer efficacy in various in vitro and animal tumor models. The effect of mebendazole on tubulin organization has been well established, and it has also been shown to interact with multiple kinases implicated in pro-tumorigenic pathways (e.g., VEGFR2, ERK, MAPK14, ABL1, etc.). While multiple kinase targets have been established, ES-Screen identified additional previously unidentified kinase targets of mebendazole. These include PIK3CG, JAK2, RAF1, and MNK2 kinases. In vitro binding assays [26] confirmed these interactions (Table 1).

#### 2.4.2. Novel NSAID Protein Targets Implicated in Metabolic Diseases

**Figure 8:**
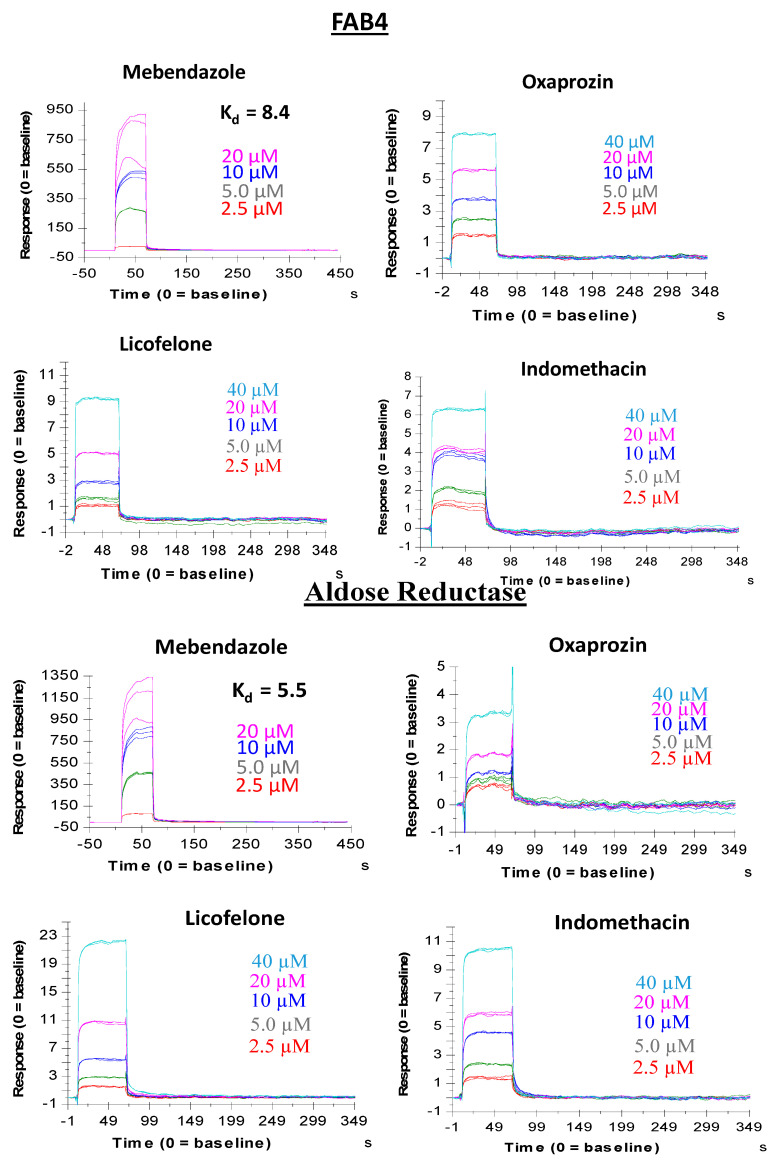
Mebendazole, Indomethacin, licofelone, and oxaprozin bind directly, in a dose-dependent manner, to immobilized human adipocyte fatty acid binding protein (FABP4) and aldose reductase (AKR1B1) in Surface Plasmon Resonance (SPR) assay. Assays were performed in triplicates for each drug with respect to each protein target.

ES-Screen identified previously unreported interactions of the non-steroidal anti-inflammatory drugs (NSAIDs) indomethacin, licofelone, and oxaprozin with human adipocyte fatty acid-binding protein (FABP4) and human aldose reductase (AKR1B1), important targets in metabolic diseases such as diabetes. We assessed the ability of these NSAIDs to bind FABP4 and AKR1B1 using Surface Plasmon Resonance (Figure 8). Indomethacin, licofelone, and oxaprozin interacted with FABP4 and AKR1B1 in a dose-dependent manner. Indomethacin produced sensorgram patterns for FABP4 and AKR1B1, where relative response units were similar across both targets with respect to the drug concentration being tested. Similar sensorgram patterns were also found for licofelone with respect to FABP4 and AKR1B1 (Figure 8). Interestingly, sensorgrams for oxaprozin differed between the two targets—relative response units were higher at each concentration of oxaprozin tested for FABP4 than AKR1B1 (Figure 8). This is consistent with ES-Screen rankings of oxaprozin for each target, with the drug ranked #16 and #19 for FABP4 and AKR1B1, respectively. Notably, for the indomethacin, the ES-Screen ranked #4 for AK1B1 but not in the top 40 for FABP4, whereas licofelone was ranked #4 for FABP4 but was not ranked in the top 40 for AKR1B1. In addition, all three NSAIDs are known inhibitors of cyclooxygenase-2 (COX-2). As a proof-of-concept, ES-Screen was tested for its ability to enrich these drugs within the top 40 rankings. When using the reference ligand-target dyad of sulindac, bound to COX-2, ES-Screen ranked indomethacin and oxaprozin as #27 and #26, respectively, whereas licofelone was not ranked within the top 40. Additionally, ES-Screen identified mebendazole binding to FABP4 and AKR1B1, and our in vitro experiment verified its binding with a KD_50_ of 8.4 µM and 5.5 µM, respectively (Figure 8). 

## 3. Discussion

Proper treatment of electrostatic interactions is critical for accurately modeling biochemical interactions. This is evident in biological processes of high fidelity and discrimination, such as in ATP- and GTP-binding proteins. Ligand-protein target interactions of biological significance are also governed by electrostatics. In drug discovery, virtual screening strategies for identifying novel ligand-protein target interactions have evolved and become exceedingly diverse. Methods such as docking have been extensively used to calculate the free energies of binding (DeltaG) and relate them to binding affinities. However, the calculation of DeltaG differs across methods by how they treat solvent effects, atomic partial charges, and sampling of conformational space, among other parameters. For instance, MM-GBSA and MM-PBSA methods incorporate molecular mechanics and implicit/explicit solvent energy functions (generalized Born and Poisson-Boltzmann with surface area solvation, respectively) for increased accuracy, though at a greater computational expense than a simple docking protocol occurring in vacuo. When solvation effects are incorporated in simulations, the polar solvation energy term represents electrostatic interactions between solute (protein/ligand) and solvent continuum. Polar solvation energy is obtained by numerically solving the PB or GB equations. Moreover, calculating electrostatic/polar contributions to free energy of binding requires simulating the protein target alone, the ligand alone, and the complex en vacuo and solvated and finally calculating the difference between complex and unbound forms. Such computationally expensive methods, while suitable for a small set of chemical congeners and predictive structure-activity relationship (SAR) studies, are not amenable to high-throughput virtual screening (HTVS) campaigns. 

Methodology in calculating the electrostatic contribution to binding free energy is critical in virtual screening paradigms. Calculations typically treat electrostatics as Coulombic interactions where the electrostatic free energy is the summation of pairwise interactions between all atoms in the system when treated as simple point charges within a homogenous medium with a uniform dielectric. More advanced modeling of electrostatic methods such as Particle Mesh Ewald (PME) to account for inhomogeneous mediums and distance-dependent dielectrics are used in molecular dynamics (MD) simulations and high-resolution modeling studies focusing on single systems but are not used in virtual screenings due to high computational cost.

In this study, we have introduced ES-Screen (Figure 1), a novel electrostatics-driven virtual screening method that leverages information from crystallized interactions and is applicable across diverse protein target systems. While ES-Screen is a holistic virtual screening strategy, its novelty lies in its uniquely explicit treatment of electrostatic interaction energies. As previously noted, energy calculations in virtual screenings typically employ Coulomb’s Law in vacuo, under implicit solvation (GBSA/PBSA) or using explicit solvation (thousands of bulk water solvent atoms considered in the calculations). As such, the total energy is regarded as an integral of the energies from all pairs of atom partial charges in the system modeled. Our method differs in that the electrostatic interaction energy is defined by the protein’s electrostatic force exhibited on ligand atoms. Therefore, it is explicitly concerned with protein-originating electrostatic potentials at the exact location of ligand atom partial charges (Figure 1B). Hence, the electrostatic interaction energy is the summation of all the products of partial charge and electrostatic potential at ligand atom-occupied sites (Equation (5)). This modeling of electrostatic interaction energy was previously shown to finely discriminate between ATP and GTP when cognate/non-cognate replacement energies were considered [1]. Given this fidelity, we provided preferential weightage to the electrostatic interaction energy term over the nonpolar and van der Waal’s contributors (Equation (6)).

The ability of ES-Screen to prioritize known binders over decoys was demonstrated for a diverse set of 10 protein targets using the DUD-E benchmarking dataset (Figure 2). It was superior to traditional docking and more rigorous calculations that account for solvation (MM-GBSA/MM-PBSA). This benchmarking is critical as this database contains structurally diverse decoy sets that maintain physicochemical descriptor similarities with the active molecules. For example, many decoys have similar net charges to the actives. Yet, ES-Screen can discriminate between them by modeling the individual atomic point charges oriented within the binding pocket with a particular interacting pattern. When ES-Screen was applied to over 50 protein targets in a protocol mimicking typical virtual screenings, ES-Screen also performed best across all methods (Figure 3 and Figure 4). A closer inspection of ES-Screen performance across protein target classes revealed the starkest difference in performance within the kinase family (Figure 5). Kinase binding sites are well defined but extensively exposed to solvent. Conversely, performance was most similar for nuclear receptors containing buried pockets with dominating hydrophobic interactions. We attribute this to differing dominant components contributing to binding—buried pockets lined by hydrophobic residues tend to have nonpolar interactions dominating binding. In contrast, more superficial pockets lined by hydrophilic residues and directly facing aqueous solvent will consequently contribute more by electrostatic interactions. In the next iteration of ES-Screen, we believe adding a correction term for the nature of the pocket (e.g., buried vs. superficial, polar vs. nonpolar residues) will minimize discrepancy in performance across protein target families.

Special attention must also be given to the G protein-coupled receptors (GPCRs) family. Briefly, GPCRs are membrane-bound proteins with flexible extra- and intracellular loops and a highly conserved transmembrane domain of seven alpha-helices. Ligands that modulate GPCR function occupy the 7-TM region in a highly variable manner, allowing them to act as inhibitors, agonists, and partial and biased agonists. ES-Screen performance exhibited the greatest variability for the GPCR set (Figure 5). Visual inspection of ligand binding poses found that poses were not optimal compared to the cognate reference ligand binding mode. Given that poses were generated via a pharmacophore method, poor poses may be attributed to the following: (1) sub-optimal scoring function by a given pharmacophore searching algorithm, (2) incorrect initial pharmacophore model generated by the experimenter, or (3) not providing constraints for atom types as multiple atom types can satisfy a general pharmacophoric criterion. In addition, the bad pose problem is magnified in GPCRs since the pocket is large relative to other protein families. When considering these together, many poses that fit the criteria imposed by scoring functions and excluded volumes can be generated. Still, the best pose from this method may not necessarily reflect the actual binding mode. We believe that the binding pose problem also reflects the counter-intuitive outcome of docking performing better than MM-GBSA/MM-PBSA for our dataset (Figure 6).

Discrimination of actives versus inactive using predefined datasets, though useful for benchmarking, does not reflect the translational pharmacology on biological systems. From the ES-Screen virtual screenings noted above on the various protein targets using FDA-approved and experimental drugs, we sought the capability of ES-Screen as an efficient strategy for drug screening. The anti-hookworm drug mebendazole (MBZ) has received much attention for its ability to be repurposed for multiple cancers. While MBZ is an inhibitor of hookworm tubulin, it demonstrates a crossover effect on mammalian tubulin [21]. It has been shown to interact with various other targets to provide synergistic tumor cell inhibition or death [23]. In addition, decades of MBZ use in the clinic suggest a favorable toxicity profile relative to current chemotherapeutics. ES-Screen identified previously unreported targets for MBZ, including PI3KCG, JAK2, RAF1, MNK2, FAB4, and Aldose reductase, with later two targets also being used identified as new targets for drugs licofelone, indomethacin, and oxaprozin and all of which were confirmed with in vitro binding assays (Table 1 and Figure 8). The association of MBZ with MNK2 is particularly interesting as MAPK-interacting kinases (Mnks) are emerging as novel oncologic targets [27,28] and lack the dearth of structural detail that many other kinases have [29]. Thus, while the current pharmacological repertoire of Mnks inhibitors is currently underdeveloped, the confirmed interaction between MNK2 and MBZ can serve as a foundation for future structural studies or the development of pharmacophores to identify other drugs or chemotypes. 

While ES-Screen performs very well, some refinements are required. The problem of the initial binding pose is critical as it propagates throughout subsequent calculations. A potential circumvention is introducing a “consensus approach” [26] to the virtual screening paradigm where multiple binding poses can be generated using either a pharmacophore- or docking-based approach without regard for scoring function and then running ES-Screen for each pose. As ES-Screen is an electrostatics-driven method, we also anticipated performance trends to correlate with protein and reference-ligand physicochemical properties that influence electrostatics (Figure 7). Interestingly, slight but non-significant trends were found for ligand properties that reflect molecular size (i.e., surface area, weight, and volume). We believe this is due to an increased number of contact points between the reference ligand and protein, resulting in more favorable binding free energies. A slight trend was also found for the number of donor/acceptor hydrogen bonding of the reference ligand, implying the greater contribution of electrostatics toward binding. Nonetheless, no significant correlations were obtained for those and other descriptors. The lack of trends for properties such as protein or ligand dipole, presence of metal cations, and protein size is also interesting, given their influence on electrostatic properties. Collectively, these outcomes suggest that ES-Screen performs accurately irrespective of variation in physicochemical properties, thus enabling it as a potential ubiquitous virtual screening tool. However, we acknowledge that this analysis is not universal as there was a difference in performance between protein target families though generally superior to docking and MM-GBSA/MM-PBSA. As noted above, a correction term with respect to binding site exposure to solvent (e.g., buried or superficial) and characteristics of the residues (e.g., polar or nonpolar) may improve outcomes and reduce the discrepancy in performances within and across protein target families. Energy terms in ES-Screen also lack molecular mechanics (e.g., energy due to intermolecular bonds, rotations, vibrations, etc.). Although ES-Screen does not attempt to recapitulate calculations of the true free energy of binding (DeltaG) as the electrostatic and nonpolar energy contributors are decomposed and separately modeled with weightage given to electrostatic interaction energy, the addition of energy due to molecular mechanics may provide further refinement.

ES-Screen is a novel electrostatic-driven knowledge-based virtual screening method that surpasses conventional methods through unique modeling of electrostatic interactions and the addition of individual replacement energy terms with respect to the cognate ligand. It is widely applicable to diverse protein targets and suitable for high-throughput virtual screenings, unlike rigorous methods such as MM-GBSA/MM-PBSA, which typically require intensive calculations through iterative molecular dynamics and, at times, explicit solvent. Furthermore, in its current form, ES-Screen does not attempt to correlate results with binding affinities but rather simply has a ranking schema that prioritizes ligands for pharmacological testing with respect to a reference cognate ligand. 

## 4. Materials and Methods

### 4.1. Ligand and Protein Target Structure Preparation

X-ray crystal structures containing ligands with <2.5Å resolution were obtained from RCSB [30]. A list of protein target names and corresponding PDB identification codes are shown in Table S1. All non-protein atoms (i.e., waters, cofactors, ligands) were removed except metal ions participating in binding. The “Protein Prep” utility in Schrodinger was then used to add explicit hydrogens, fix disulfide bonds, and correct protonation states at pH 7.0.

Reference molecules for corresponding protein crystal structures were downloaded from RCSB. Structures were manually corrected in Schrodinger to maintain crystallized conformation, bond order, formal charge, and protonation states. Benchmark active and decoy molecules for a subset of protein targets were obtained from the DUD-E [17] and not modified further. Structures were collected from BindingDB [31] and DrugBank [32] for the targets being assessed with FDA-approved and experimental drugs. Respectively known drug binders were also obtained from DrugBank and RCSB. Drugs with a molecular weight < 750 g/mol were subsequently subjected to Schrodinger’s LigPrep [33] program to convert three-dimensional energy-minimized structures at pH 7.0 for physiological protonation states. The final drug dataset for performance assessment and downstream drug repurposing included 3601 drugs.

### 4.2. Comparative Docking

Schrodinger Glide-SP [34] was used to dock ligands into protein target binding sites. Glide was chosen as it is known for accurately predicting binding geometries. 20 Å grids around the centroids of co-crystallized reference molecules were used to identify the binding sites. Glide docking with 10-pose minimization and remaining default settings were used to simulate true virtual screening experiments of large chemical databases. Docking scores corresponding to the free energy of binding ($\Delta G_{binding}$) were extracted for each ligand.

### 4.3. ES-Screen

ES-Screen is an electrostatics-driven multi-modular method that culminates in calculating a comprehensive Z-score used for ligand ranking with respect to each target (Figure 5A). Individual steps are explained below.

#### 4.3.1. Pharmacophore Screening

Phase [35] pharmacophore screening was used to place query ligands into target pockets with proper orientations of functional groups contributing to the binding. Pharmacophore hypotheses were constructed manually for every target based on visual inspection of the reference interactions using PoseView from the RCSB website. Excluded volumes of binding site atoms, defined as 10 Å from the geometric center of the bound reference molecule, were integrated to filter for ligands of appropriate size and that can adopt conformations that fit the defined pocket shape while maintaining the necessary functional group interactions. The use of excluded volumes also helped avoid clashes between atoms of the query ligands and the protein targets. A minimum of 3 pharmacophore matching sites was required for survival, and the best pose was determined by a “fitness score” equation implemented in Phase. Surviving ligand poses were retained for subsequent steps, and “fitness scores” were discarded.

#### 4.3.2. Calculation of Electrostatic Energies of Interaction

Gasteiger partial charges were assigned to ligand atoms using Antechamber [36,37] within AmberTools 16 [38]. DelPhi [39] was used to calculate the ligand-free electrostatic potential (ESP, *Φ*) contributed by the protein at each ligand atom coordinate within the protein binding site using the linearized Poisson-Boltzmann Equation for implicit solvation. DelPhi was chosen for its high accuracy in treating multivalent ions, irregular protein shapes, and multiple dielectric constants. All ligands and heteroatoms except structural and catalytic metal ions were removed. Proteins were placed at the center of a cubic box, with 70% of the box edge occupying the protein’s longest Cartesian dimension. The following parameters were used: protein and solvent dielectric constants—2 and 80; ionic strength—0.145 M; ionic radius: 2.0 A; solvent probe radius: 1.4 A; grid resolution: 0.6 A/grid point. Linear interpolation of the electrostatic potential of the surrounding grid points provided binding site ESP (ϕ) values within binding sites (at each protein-bound ligand atom site). Simple cognate (reference ligand) and non-cognate (query ligand) electrostatic energies of interaction ($E_{electrostatic}$) were calculated using an in-house script using Equation (1):

(1) $$E_{electrostatic}=\sum_{j=1}^{n}\phi_{j}q_{j}$$

$E_{electrostatic}$ is formally defined as the electrostatic interaction energy from assembling ligand atom partial charges within the inhomogeneous medium (low dielectric binding site surrounded by the high dielectric solvent) from a uniform dielectric continuum (constant high dielectric solvent). It represents energies derived from the electrostatic distribution of the ligand-free protein. This energy differs from $G_{electrostatic}$, which represents the total electrostatic free energy according to Coulomb’s law:

(2) $$G_{electrostatic}=\Delta G_{solv}^{polar}+G_{coulomb}$$

where $\Delta G_{solv}^{polar}$ is the polar solvation term and $G_{coulomb}$ is the coulombic energy of the ligand-protein complex system. $G_{electrostatic}$ is generally derived from the summation of the ESP (ϕ) multiplied by the partial charge with respect to every solute atom (protein and ligand), resulting in the calculation of the total electrostatic free energy. Our method relies on the electrostatic interaction energy $E_{electrostatic}$ derived only from ligand-free protein electrostatic potentials at ligand atom-occupied sites. The process of calculating $E_{electrostatic}$ is schematically depicted in Figure 5B. 

#### 4.3.3. Calculation of Components of Non-Polar Solvation Free Energy Contributors

Hydrophobic ($E_{hydrophobic}$) and van der Waal’s ($E_{vdw}$) free energies were considered representative of the non-polar solvation free energy term ($\Delta G_{solv}^{non-polar}$). Prime MM-GBSA [40] within the Schrodinger software suite was used to calculate $E_{hydrophobic}$ and $E_{vdw}$ for ligand-protein complexes. Default settings were kept with no protein flexibility to represent calculations of single-point energies. 

#### 4.3.4. Calculation of Replacement Energies of Interaction

Replacement energies are the energy costs of placing a query (non-cognate) ligand at the binding site in place of the reference (cognate) molecule. It is formally represented as:

(3) $$\Delta E_{cognate\to noncognate}=E_{noncognate}-E_{cognate}$$

where $E_{cognate}$ and $E_{noncognate}$ represent simple electrostatic ($E_{electrostatic}$), hydrophobic ($E_{hydrophobic}$) or van der Waal’s ($E_{vdw}$) interaction energies. 

As simple electrostatic interaction energies are based on only ligand-free protein electrostatic potentials, simple electrostatic replacement energies ($\Delta E_{electrostatics}$) do not reflect the total electrostatic cost of replacement. The full cost is comprehensively captured using $\Delta \Delta G_{electrostatic}$, the difference in the electrostatic free energy of binding:

(4) $$\Delta \Delta G_{electrostatic}=\Delta G_{noncognate}-\Delta G_{cognate}$$

However, our goal is not to achieve exact values for binding free energies but rather to relatively quantitate binding potential to prioritize ligands. We have previously shown that differences in $\Delta E_{electrostatics}$ for the replacement of adenine by guanine and the converse with respect to their specific binding proteins implicitly suggest a similar difference in $\Delta \Delta G_{electrostatic}$ and is sufficient for adenine/guanine molecular discrimination by those proteins [1,2]. Thus, we expect $\Delta E_{electrostatics}$ to reflect the electrostatic component of the binding potential for query (non-cognate) ligands with respect to the reference (cognate). We extend this assumption for the non-polar contributions by calculating replacement energies $\Delta E_{hydrophobic}$ and $\Delta E_{vdw}$. Negative replacement energies (ΔE<0) are energetically favorable for binding of the query ligand with respect to the given protein conformation and binding state. 

Each replacement energy term ($\Delta E_{electrostatics}$, $\Delta E_{hydrophobic}$, and $\Delta E_{vdw}$) was then normalized as follows: if ΔE≤0, then the values are normalized to a value of 1 to reflect favorable binding of the query ligand relative to the reference; if ΔE>0, then it is subjected to the normalization procedure explained below. Utilizing replacement energies for individual contributors to the free energy of binding enriches ES-Screen outcomes as it optimizes over the individual components and provides finer granularity.

#### 4.3.5. Physicochemical Descriptor Generation and Similarity Scoring

Protein target-normalized dipole moments were calculated using dipol server [41]. QikProp [42] was used to generate the ligand-centric descriptors. The following descriptors were used: (1) number of hydrogen-bond acceptors, (2) dipole moment, (3) number of hydrogen-bond donors, (4) electron affinity, (5) globularity, (6) ionization potential, (7) solvent-accessible surface area (SASA), and (8) volume. Strike [43] was used to calculate atom pair similarity and similarities of the other ligand-centric descriptors to the reference molecule. Similarities were quantified as Tanimoto coefficients (T_c_) on the 0–1 unit range, with T_c_ = 1 representing maximal similarity and T_c_ = 0 representing least similarity. 

#### 4.3.6. Shape Quantification of Ligand/Protein Binding Pockets and Shape Similarity

Three-dimensional shape quantifications were calculated using spherical harmonics expansion coefficients via Java software provided to us by the Thornton group [44]. Post-pharmacophore screening poses were extracted before ligand shape calculations. For protein binding site shape, the ligand-occupied volume of the pocket was first identified by residues within 10 Å of the reference molecule’s centroid, followed by protomol generation using Sybyl X.1 (Certara, Inc., NJ, USA) to fill the volume with carbon and hydrogen atom probes. Protomol PDB files were then subjected to spherical harmonics expansion to quantify the ligand-occupied binding site shape. Euclidean distances were then calculated using the same Java software to quantify ligand-to-reference and ligand-to-binding site shape similarities, where smaller Euclidean distances represent greater similarity.

#### 4.3.7. Normalization Procedure

Unit scales differ between replacement energies of interaction and shape Euclidean distances, thus requiring a specialized normalization function that preserves the information before implementing it into the Z-score ranking equation. We developed an in-house script to normalize these values onto the 0–1 unit range using the sigmoid function:

(5) $$N_{\alpha }(x)=1-|1-S_{\alpha }(x)|$$

where *x* is the raw parameter, *S(x)* is a sigmoid function defined as the hyperbolic tangent function, and *α* is a tunable scalar coefficient chosen to maximize the information-preserving variance in the image of Equation (4). Since the range varied significantly between parameters, the coefficient varied as well. *α* was set to 0.05 for $\Delta E_{hydrophobic}$ and $\Delta E_{vdw}$, whose values were large magnitude and represented as kJ/mol. For $\Delta E_{electrostatic}$ (kcal/mol) and shape Euclidean distances, whose magnitudes were much smaller and ranges corresponded well with each other, α was set to 0.25.

#### 4.3.8. Z-Score Ranking Equation

The aforementioned components are combined into a Z-score for ligand ranking using the following equation:

(6) $$Z(q,p,r)=\omega_{E}\Delta E_{electrostatic}+\omega_{H}\Delta E_{hydrophobic}+\omega_{vdw}\Delta E_{vdw}+\sum_{m=1}^{M}[\omega_{m}f_{m}(p,q)+\omega_{m}^{\prime }f_{m}^{\prime }(r,q)]+\sum_{n=1}^{N}X_{n}(r,q)+\omega_{A}AP(r,q)$$

where *q, p* and *r* correspond to the query ligand, protein target, and reference molecule, respectively. $\Delta E_{electrostatic}$, $\Delta E_{hydrophobic}$, and $\Delta E_{vdw}$ correspond to the electrostatic, lipophilic, and van der Waal’s replacement energies with their respective weights ($\omega_{E}$ = 2, $\omega_{H}$ = 1, $\omega_{vdw}$ = 1). The first summation corresponds to the shape similarity metric composed of two functions: (1) $\omega_{m}f_{m}(p,q)$, where $f_{m}$ is the shape function corresponding to a similarity quantification between the pocket shape of the protein target *p* and the query ligand *q* with weighting factor $\omega_{m}$ = 1, and (2) $\omega_{m}^{\prime }f_{m}^{\prime }(r,q)$, where $f_{m}^{\prime }$ is a shape function corresponding to a similarity quantification between reference shape *r* and query ligand shape *q* with weighting factor $\omega_{m}^{\prime }$ = 2. Normalized Euclidean distances are used to quantify shape similarity. The second summation term corresponds to the combined similarity of *N* = 8 query ligand-based descriptors terms ($X_{n}$) to reference *r* using normalized T_c_ scores.AP(r,q) corresponds to the Tanimoto coefficient for the atom pair similarity between reference and query ligands with weight $\omega_{A}$ = 4. Ligands with the highest Z-scores are predicted most likely to bind to the protein target.

### 4.4. MM-GBSA/MM-PBSA Binding Energy Calculations

Molecular mechanics-Generalized Born (GB) and Poisson-Boltzmann (PB) surface area methods were used to calculate the free energies of binding ($\Delta G_{binding}$) under implicit solvent conditions for putative ligand-protein target complexes after pharmacophore placement. MM-GBSA calculations were conducted in Schrodinger, as noted above, with default parameters and no protein flexibility. These settings were used to calculate energies and to avoid the exponential computational costs of incorporating molecular dynamics in the calculations, thus simulating a virtual screening protocol. 

MM-PBSA calculations were performed using the Python script MMPBSA.py packaged with the AmberTools16 suite. Gasteiger atom partial charges were first generated using Antechamber. Topology and coordinate files of the ligands, protein targets, and complexes were subsequently generated using the FF14SB and GAFF2 forcefields through the LEaP module in AmberTools16. Phosphorylated protein residues were modeled using parameters found in the phosaa10 data file packaged with AmberTools. For protein targets containing metal cations, ions were parameterized via the 12-6 Lennard-Jones nonbonded model. The bonded model was foregone as no molecular dynamics simulations were conducted. The MMPBSA.py script was then run on the generated topology and coordinate files with the following input parameters: istrng = 0.145, indi = 2.0, exdi = 80.0. These values correspond to the physiological ionic strength in Molarity, internal dielectric constant, and external dielectric constant, respectively. $\Delta G_{binding}$ values were then extracted from the output files.

### 4.5. Performance Metrics

ES-Screen, GLIDE-SP and MM-GBSA/PBSA virtual screening performances were initially assessed using areas under the Receiver Operating Characteristic (ROC) curves [45]. However, since practical virtual screenings in drug discovery experimentally test only a small fraction of ligands, the Enrichment Factors (EF) is used to measure early performance. We mainly utilize relative EFs because general EFs are influenced by the number of actives in a dataset [46]. Relative EF is defined as:

(7) $$EF_{rel}=\frac{n_{a}}{min(N_{x\%},n)}\times 100$$

where *x*% corresponds to a defined cutoff (i.e., top 1% of the database), *N* is the total number of molecules in the database, *n_a_* is the number of actives found in the top *x*%, and min(*N_x_*_%_,*n*) is the maximum number of actives that can be found in the top *x*% (i.e., maximum possible enrichment). We report relative EFs for the top 1% and 5% as the greatest false positivity rates in virtual screenings tend to occur in this region of databases. 

### 4.6. FABP4 and Aldose Reductase Surface Plasmon Resonance (SPR) Binding Kinetics Assay

The determination of Mebendazole (MBZ), Licofelone, Indomethacin, and Oxaprozin binding kinetics (K_d_) to FABP4 and Aldose Reductase predicted by ES-Screen was performed by surface plasmon resonance experiments with a Biacore T100 equipped with a CM5 sensor chip as described by us previously [47,48]. Briefly, mouse extracellular domain 1–2 (EC 1–2) C-terminally cysteine-tagged FABP4 and Aldose Reductase recombinant protein was immobilized on flow cell (FC) 2 in HEPES buffered saline (10 mM Hepes, pH 7.4, and 150 mM NaCl, 3 mM CaCl_2_) using a thiol-coupling kit according to the manufacturer’s protocol, resulting in immobilization levels of 4580 response units (RU). FC1 was only activated and inactivated and used as a reference. Mebendazole, Licofelone, Indomethacin, and Oxaprozin stock solution was diluted to a final concentration of 200, 100, 50, 25, 12 μM and injected in 10 mM Hepes, 150 mM NaCl, 3 mM CaCl_2_, 1% DMSO, and 0.5% P20. Each injection was repeated three times for 60 s. FC1 signals were deducted from FC2 for background noise elimination.

### 4.7. Kinase Binding Assay

Kinase assays were performed using Kinomescan, by Discoverx, CA, USA and Caliper LabChip 3000 by Caliper Life sciences, USA as described by us previously [47,48]. The determination of Mebendazole (MBZ) thermodynamic binding affinities (K_d_) to kinase targets predicted by ES-Screen was performed by using active site-directed competition binding [49]. Kinase-tagged T7 phage strains were grown in parallel in 24-well blocks in an E. coli host derived from the BL21 strain. E. coli bacteria were grown to log-phase and infected with T7 phage from a frozen stock (multiplicity of infection = 0.4) and incubated with shaking at 32 °C until lysis (90–150 min). The lysates were centrifuged (6000× *g*) and filtered (0.2 μm sieves) to remove cell debris. The remaining kinases were produced in HEK-293 cells and subsequently tagged with DNA for qPCR detection. Streptavidin-coated magnetic beads were treated with control (biotinylated) for 30 min at room temperature to generate affinity resins for kinase assays. The liganded beads were blocked with excess biotin and washed with blocking buffer (SeaBlock (Pierce), 1% BSA, 0.05% Tween 20, 1 mM DTT) to remove unbound ligands and to reduce non-specific phage binding. Binding reactions were assembled by combining kinases, control liganded affinity beads, and mebendazole in 1× binding buffer (20% SeaBlock, 0.17 × PBS, 0.05% Tween 20, 6 mM DTT). Mebendazole was prepared as 40× stocks in 100% DMSO and directly diluted into the assay. All reactions were performed in polypropylene 384-well plates in a final volume of 0.04 mL. The assay plates were incubated at room temperature with shaking for 1 h and the affinity beads were washed with wash buffer (1 × PBS, 0.05% Tween 20). The beads were then re-suspended in elution buffer (1 × PBS, 0.05% Tween 20, 0.5 μM non-biotinylated affinity ligand) and incubated at room temperature with shaking for 30 min. The kinase concentration in the eluates was measured by qPCR. Ligands that bind the kinase active site and directly prevent kinase binding to the immobilized ligand will reduce the amount of kinase captured. In contrast, ligands that do not bind the kinase have no effect on the amount of kinase captured. The amount of kinase captured in test versus control samples were measured by using a quantitative, precise and ultra-sensitive qPCR method that detects the associated DNA label. Using the primary screen binding interactions are reported as ‘% Ctrl’ (Percent kinase remaining activity, Equation (8)), where lower numbers indicate stronger hits.

(8) $$\mathrm{Percent}\;\mathrm{Control}\;(\%\mathrm{Ctrl})=\frac{\mathrm{Mebendazole}\;\mathrm{signal}\;-\;\mathrm{Positive}\;\mathrm{control}\;\mathrm{signal}}{\mathrm{DMSO}\;\mathrm{Negative}\;\mathrm{control}\;\mathrm{signal}\;-\;\mathrm{Positive}\;\mathrm{control}\;\mathrm{signal}}\;\times \;100$$

In a similar manner, binding constants (K_d_) for mebendazole-kinase interactions are calculated by measuring the amount of kinase captured as a function of the mebendazole concentration in a dose response manner. An 11-point 3-fold serial dilution of each test compound was prepared in 100% DMSO at 100× final test concentration and subsequently diluted to 1× in the assay (final DMSO concentration = 1%). Most K_d_s were determined using a starting concentration = 30,000 nM. If the initial K_d_ determined was <0.5 nM (the lowest concentration tested), the measurement was repeated with a serial dilution starting at a lower starting concentration. Binding constants (K_d_) were calculated with a standard dose–response curve (ligand dose (*x*-axis)—qPCR signal (*y*-axis)) using the Hill Equation (9), with the Hill Slope set to −1.

(9) $$\mathrm{Response}\;(Y)=\mathrm{Background}+\frac{\mathrm{Signal}(\max)\;-\;\mathrm{Background}}{1+(\frac{k_{d}}{\mathrm{Drug}\;\mathrm{Dose}\;(X)})\;\mathrm{Hill}\;\mathrm{Slope}}$$

## 5. Conclusions

We have developed ES-Screen, a novel electrostatics-driven virtual screening method for predicting ligand-target interactions for drug discovery and repurposing. ES-Screen capitalizes on experimentally determined ligand-target signatures from crystal structures, rendering it a knowledge-based approach. As electrostatic interactions are critical for biomolecular associations [1,2,3,4,5,6,7,8,50,51], ES-Screen explicitly models and prioritizes electrostatic interactions in its ranking equation. In addition, the use of replacement energies with respect to a reference cognate ligand optimizes for ligands energetically more favorable for binding than the reference with respect to both polar and non-polar interactions. The use of replacement energies and prioritizing electrostatic contributions in energy calculations make ES-Screen a first-in-class virtual screening method. ES-Screen performs well across protein target systems with diverse physicochemical properties, implying potential use for any potential protein target in perspective virtual screenings. Compared to traditional energy calculations of ligand-target complexes that rely on molecular mechanics/dynamics and solvation effects (e.g., MM-GBSA and MM-PBSA), which are computationally expensive, ES-Screen performs superiorly and is significantly more efficient. Thus, ES-Screen is more suitable for high-throughput virtual screenings. In contrast, MM-GBSA/MM-PBSA methods are more limited to a small fraction of the virtual chemical library (e.g., the top 1% derived from docking) or for small libraries of chemical congeners. ES-Screen was also used to identify previously unknown cancer-associated kinase targets for the anthelminthic mebendazole. Notably, ES-Screen identified several new targets for mebendazole, oxaprozin, indomethacin, and licofelone. Therefore, ES-Screen may serve as a valuable tool in pre-clinical drug development for discovering novel ligand-target interactions with high accuracy and efficiency. This electrostatics-driven approach applicability is diverse in nature, extending from ligand binding prediction to lead optimization studies such as differentiating active vs. non-active and high to low potent among similar ligands, which we are already progressing successfully. The other applications include hard-to-dock: metal-ion binding to protein, protein to protein, protein to an antibody, antibody to an antigen, and simulating cellular environments.

## 6. Limitations

We discuss here the limitations of the current version of the ES-Screen. It is seen that ES-Screen has many other components in addition to the major electrostatic component. As such, if the ES-Screen method is compared with GBSA or PBSA, or other components, it is unclear whether the effect is due to the new ES-Screen or the other components. To explore that and as can be seen in methods Section 4.3.3 and note that in Figure 4, we performed and showed an incremental increase in performance after the inclusion of ES-Screen. Further, ES-Screen uses pharmacophore-based docking in which ligands are first placed into putative binding pockets through knowledge-based pharmacophore models. Such models are derived from crystal structures to optimize ligand poses by shape and functional group orientation. In the case, if the protein does not have any co-crystalized ligand, how the energy is calculated is a limitation of ES-Screen in its current version. We noted this in the discussion section. Furthermore, ES-Screen does not attempt to correlate results with binding affinities but rather simply has a ranking schema that prioritizes ligands for pharmacological testing with respect to a reference cognate ligand. 

The ES-Screen selects the best hit compounds based on the Z score, which may need to be more reliable for the binding affinity predictions. The ES-Screen Z-score is not meant to correlate directly with binding affinity. To determine the reliability of the ES-Screen predictions, we assessed performance by looking at Enrichment Factors (EF1% & EF5%) for the DUD-E benchmarks (Figure 3). We then did a prospective testing of ligands highest ranked for certain protein targets based on their Z-score. This is detailed in Section 2.4, Experimental Validation of ES-Screen Predictions. 

Our aim here is to implement this unique electrostatics-driven virtual screening approach. To explore the success of this approach in a rapid way and as a proof-of-concept, we used commercial software initially. For obvious reasons, we may not be able to make ES-Screen available in its current form to download, as it involves commercial software. Though some of the modules of ES-Screen utilize commercially available software, this software can be exchanged with open-source software across multiple operating systems, thus making ES-Screen feasible for many investigators across both academic and industrial institutions and widely employable by the entire research community. Further, as the next step in progress, we are updating the first version of the ES-Screen and addressing the limitations. Currently, with the aid of publicly available free software, we plan to develop a web server to run the ES-Screen online. In the second version, we aim to improve and make further enhancements to the ES-Screen by introducing protein dynamic flexibility and minimizing the complexity by reducing the combination of several other parameter components using the free software. Notably, we are making the ES-Screen a purely electrostatics-driven method (standalone) for lead identification and optimization. We further aim to develop a web server by integrating open-source software that further simplifies the process for novice investigators for further community engagement.

## Figures and Tables

**Table 1:** Kinase binding assay of MBZ for predicted kinase hits.

Kinase Target	Percent Control at 10 µM (Lower Numbers Indicate Stronger Hits)	Binding Affinity (K_d_) in nM
JAK2	39	N/D
PIK3CG	18	N/D
MKNK2	46	N/D
RAF1	23	N/D

## Data Availability

The data presented in this study are available upon request from the corresponding author.

## Supplementary Materials

The following supporting information can be downloaded at: https://www.mdpi.com/article/10.3390/ijms232314830/s1.
