# Supplementary material for: ES-Screen: A Novel Electrostatics-Driven Method for Drug Discovery Virtual Screening
Source: Int J Mol Sci. 2022 Nov 27;23(23):14830. doi: 10.3390/ijms232314830 (PMC9736079; doi:10.3390/ijms232314830)
Supplement: Supplementary file 1 [file ijms-23-14830-s001.zip › ijms-1969854-supplementary.pdf]

Table S1. Protein targets studied are listed by PDB IDs and gene symbols.

PDB ID	Gene Symbol	PDB ID	Gene Symbol
		3ERD	ESR1
1ONY	PTP1B	3GBS	PNPH
3D4Q	BRAF	3FRJ	HSD11B1
4KZ0	PIK3CG	2NNQ	FABP4
3RX3	ALDR	1MZN	RXRA
3SFF	HDAC8	4KUW	CA2
2P2H	VEGFR2	1UK1	PARP1
4N6H	OPRD	3VEV	HXK4
2RGP	EGFR	2ICA	ITAL
3LPB	JAK2	3VSO	PPARG
3CCW	HMDH	3QKL	AKT1
2I78	DPP4	3FMK	MAPK14
3LN1	COX2	3VF3	BACE1
3KBA	PRGR	3L3Z	AR
1YPE	F2	2P54	PPARA
3KL6	F10	3EML	AA2AR
1DB1	VDX	3CJO	KIF11
1L2S	AMPC	3R3Z	HRH1
4NTJ	P2Y12R	2OJG	ERK2
4ACC	GSK3B	3FW3	CA4
2W3A	DHFR	4P6X	GRL
3NYA	ADRB2	4FUG	PLAU
1UDT	PDE5A	2HYY	ABL1
4JVR	MDM2	3LGP	ADAM17
4O05	HSP90A	1JJ9	MMP8
4EY7	ACES	3I7G	MMP13
1ZW5	FPPS	1O86	ACE
